# Clinical and epidemiologic characteristics associated with dengue fever in Mombasa, Kenya

**DOI:** 10.1016/j.ijid.2020.08.074

**Published:** 2020-11

**Authors:** Jacqueline Kyungah Lim, Sultani Hadley Matendechero, Neal Alexander, Jung-Seok Lee, Kang Sung Lee, Suk Namkung, Esther Andia, Noah Oyembo, Sl-Ki Lim, Henry Kanyi, So Hee Bae, Jae Seung Yang, Mary A. Ochola, Tansy Edwards, In-Kyu Yoon, Sammy M. Njenga

**Affiliations:** a International Vaccine Institute, Seoul, Republic of Korea; b Communicable Disease Prevention and Control, Ministry of Health, Nairobi, Kenya; c Faculty of Epidemiology and Population Health, London School of Hygiene and Tropical Medicine, United Kingdom; d Eastern and Southern Africa Centre of International Parasite Control (ESACIPAC), Kenya Medical Research Institute (KEMRI), Nairobi, Kenya; e Coast Provincial General Hospital, Mombasa County, Kenya; f Coalition for Epidemic Preparedness Innovations (CEPI), Washington, D.C., USA

**Keywords:** CI, confidence interval, CPGH, Coast Provincial General Hospital, °C, Celsius degrees, CRF, case report form, DENV, dengue virus, DF, dengue fever, DHF, dengue hemorrhagic fever, DSS, dengue shock syndrome, DVI, Dengue Vaccine Initiative, ELISA, enzyme-linked immunosorbent assay, ICF, informed consent form, IgM/IgG, immunoglobulin type M and type G, IRB, Institutional Review Board, KEMRI, Kenya Medical Research Institute, KEPH, Kenya Essential Package for Health, RDT, rapid diagnostic test, RT-PCR, reverse transcriptase-polymerase chain reaction, URI, upper respiratory illness, Dengue, Kenya, Africa, Surveillance, Children, Outbreak

## Abstract

**Objectives:**

Information on dengue in Africa is limited. To estimate the proportion of dengue-positive cases among febrile patients and describe clinical indicators of dengue, we conducted passive health facility-based fever surveillance in Mombasa, Kenya.

**Methods:**

Non-malarial febrile patients between one and 55 years were enrolled at three health facilities between March 2016 and May 2017. Acute and convalescent blood samples were collected with an interval of 10–21 days. Acute samples were tested with dengue RDT and a selected subset with RT-PCR, and acute/convalescent samples with IgM/IgG ELISA.

**Results:**

Among 482 enrollees, 295 (61.2%) were dengue-positive based on laboratory results. The surveillance covered the beginning of a dengue outbreak in April-May 2017, during which 73.9% of enrollees were dengue-positive. By contrast, during the non-outbreak period, 54.6% were dengue-positive. Dengue case status was positively associated with rash, fatigue, headache, retro-orbital pain, nausea/vomiting, nose bleeding, gum bleeding, loss of appetite, myalgia, and arthralgia. Dengue-positive cases in our study had mostly mild disease, with only two requiring observation, and no DHF.

**Conclusions:**

The clinical response was generally mild relative to what was observed in SE Asia and the Americas. Given the high level of DENV transmission in Mombasa, more data would be needed to further understand the disease burden and improve case detection for surveillance/monitoring of outbreaks.

## Highlights

• Data are lacking on dengue in Africa.
• This surveillance covered the beginning of a dengue outbreak in April-May 2017.
• 61% of 482 patients with non-malarial fever in Mombasa were dengue-positive.
• Dengue cases presented with rash, retro-orbital pain, myalgia, arthralgia, etc.
• Dengue cases were mostly mild with only two cases requiring observation, and no DHF.

## Introduction

Dengue fever (DF) is a mosquito-borne flavivirus infection caused by four related but antigenically distinct dengue viruses (DENVs; serotypes 1–4) and is a major and rapidly increasing global public health problem (Boisier et al., 1994). Recent studies have estimated an annual incidence of 50–100 million symptomatic infections globally, with 50,000 dengue hemorrhagic fever (DHF) cases requiring hospitalization and approximately 20,000 deaths annually (Bhatt et al., 2013, World Health Organization, 2009, Halstead, 1988, Gubler, 1999, Singhi et al., 2007).

Despite the documented presence of *Aedes* mosquitoes and dengue cases in Africa, most reports have come from a small number of countries, with few prospective and population-based studies (Surtees, 1967, Kamgang et al., 2013, Messina et al., 2014, Amarasinghe et al., 2011, Kraemer et al., 2015, Baba et al., 2016). With many competing public health problems, the clinical presentation of dengue is non-specific and difficult to distinguish from other causes of febrile illness, especially with dengue diagnostic assays not widely available (Were, 2012). Also, unlike many countries in Asia and Latin America, most African countries lack systems of mandatory reporting of dengue cases (Beatty et al., 2010).

In Kenya, compared to other African countries, there is some evidence on dengue, with several documented epidemics and outbreaks in different locations. The most recent outbreak reported was from Mombasa in May 2017 (World Health Organization, 2017). In 2011, an outbreak was confirmed in Mandera, North Eastern Region, and, in 2013, another in Mombasa continuing into 2014 (Ellis et al., 2015, Obonyo et al., 2018). In addition to outbreak investigations, a study based on 868 febrile patients, identified from September 2011 to December 2014 in multiple locations in Kenya, reported 40% (345/868) to be dengue-positive by either IgM enzyme-linked immunosorbent assay (ELISA) or by RT-PCR (Konongoi et al., 2016).

In terms of seroprevalence, in 2016–2017, a study conducted in rural Taita–Taveta County and urban slums of Kibera, Nairobi, tested 560 samples from febrile patients for DENV IgM, IgG, and NS1 antigen (Masika et al., 2020). The study found IgG seroprevalence to be 3.5% in Nairobi and 14.6% in Taita–Taveta, confirming local transmission in this part of rural Kenya (Masika et al., 2020). Between 2010 and 2011, a hospital-based cross-sectional survey was conducted in Western Kenya among children aged 12 years and under (Inziani et al., 2020). Of 656 children, 1%, 9%, and 20% tested positive by indirect ELISA for DENV 1, 2, and 3, respectively (Inziani et al., 2020). Also, dengue was retrospectively found to be the most common viral pathogen in HIV-negative samples from the 2007 Kenya AIDS Indicator Survey, with 12.5% having dengue IgG (Ochieng et al., 2015). Similarly, a household survey in Mombasa reported 13% with serological (IgM) evidence of either past or current DENV infection. While such information suggests a notable dengue transmission in Kenya, its magnitude remains mostly unknown (Ellis et al., 2015, Ochieng et al., 2015).

## Methods

### Site selection

**Figure 1:**
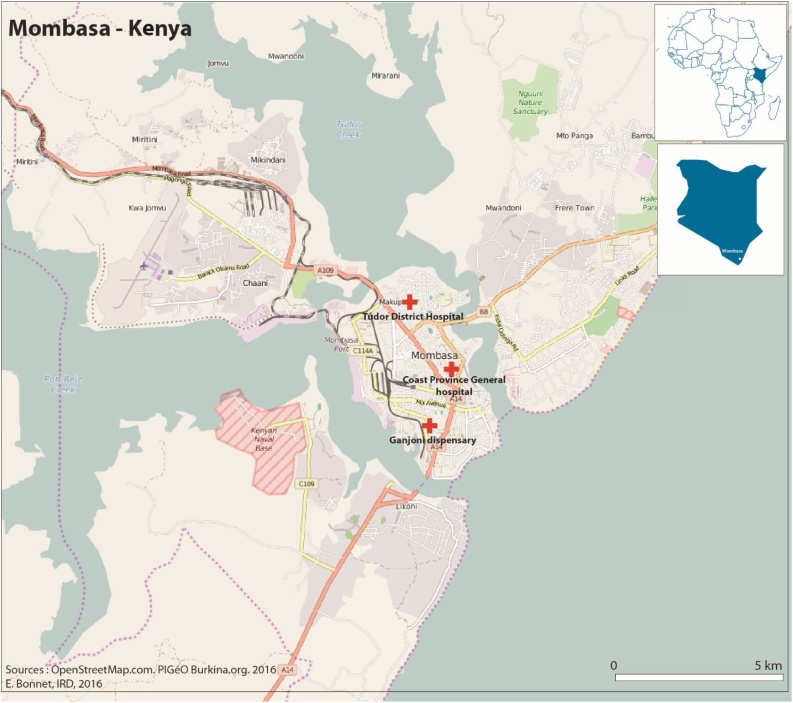
A map of the area of catchment population and study facilities. Notes: The map shows the approximate location of the three selected study facilities (Ganjoni health center, Tudor sub-county Hospital, and Coast Provincial General Hospital), covering the catchment area population of residents of Mvita sub-county, Mombasa, Kenya. Source: (Lim et al. (2018)).

Site selection was based on available data in the published literature as well as available research infrastructure (Messina et al., 2014, Lim et al., 2018, Brady et al., 2012), after consultation with local collaborators from the Kenya Medical Research Institute (KEMRI) and the Ministry of Health of Kenya. Ganjoni health center, Tudor sub-county Hospital, and Coast Provincial General Hospital (CPGH) were selected, serving a catchment population of 74,735 residents in Mombasa (Figure 1) (Lim et al., 2018, Central Intelligence Agency, 2020, Wikipedia, 2020).

In the 6-level system of healthcare service delivery in Kenya, Ganjoni health center is a Kenya Essential Package for Health (KEPH) level 2 health service provider, focusing on primary care and health promotion for the community. Tudor sub-county Hospital is KEPH level 4, district-level health center with outpatient and observation care, and CPGH is KEPH level 5, the largest tertiary referral hospital in the entire coastal region.

### Study area and population

Coastal Kenya, in eastern Africa, has a warm and humid tropical climate (Wikipedia, 2020). Mombasa has a population of about 1.3 million, of whom almost 50% are under 15 years of age (Singhi et al., 2007, Central Intelligence Agency, 2020). The “long rains” period begins around April and the “short rains” around October (Wikipedia, 2020). This study took place between March 2016 and May 2017 (15 months).

### Study design

**Figure 2:**
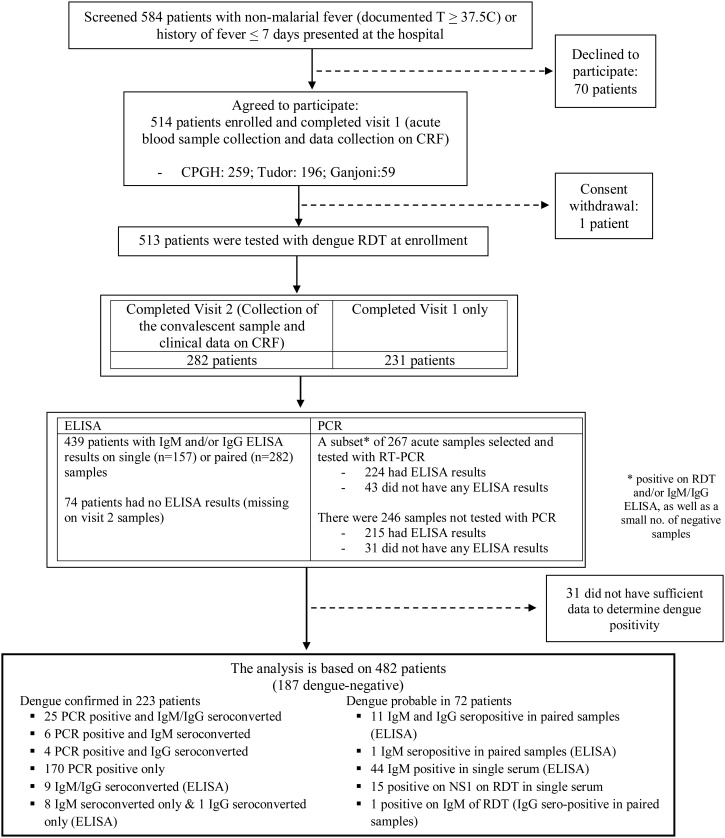
Patient flow in the passive fever surveillance at the study facilities. Notes: The chart shows the flow of patients from screening, enrollment to study participation, with determination of laboratory-based status of dengue infection, as well as how the analysis sample was reached.

Investigational methods used in this study have previously been described (Lim et al., 2018). To estimate the proportion of dengue cases among non-malarial febrile patients, and compare their clinical and epidemiologic patterns to non-dengue febrile patients, the Dengue Vaccine Initiative (DVI), in collaboration with KEMRI and the Ministry of Health of Kenya, conducted passive health facility-based fever surveillance in Mombasa, Kenya. In both outpatient and inpatient departments at the three selected facilities, patients who were febrile or with a history of fever in the past seven days were tested for malaria using RDT (either CareStart Malaria or SD BIOLINE Malaria kit) as part of routine practice. Those malaria RDT-negative patients who were eligible (see below) and agreed to participate were enrolled (Figure 2). They were tested with the SD BIOLINE Dengue Duo RDT kit, which detects both the dengue virus NS1 antigen and antibodies (dengue IgG/IgM). An acute sample of blood was taken at first presentation (visit 1). A study physician conducted interviews and physical examinations, and the surveillance case report form was completed to capture medical history, with demographic information, symptoms, and laboratory results (Lim et al., 2018).

The patient was asked to return to the facility for the convalescent sample collection 10−14 days after the first visit. After the 14th day, if the patient had not returned, a house visit was made to collect the second blood sample within 21 days of visit 1.

### Study participants

Individuals who met the following criteria were eligible for study enrollment:

1 Age 1–55 years;
2 Resident (for ≥12 months) of the catchment area;
3 Signed written informed consent from all adult subjects;
4 Written assent for those aged between 13–17 years with informed consent by a parent or guardian of any child participant;
5 Patients presenting with current fever (body temperature ≥37.5 °C) or history of fever for ≤7 days duration without localizing signs (fever caused by a localized infection or with known etiology other than dengue, such as malaria-positive by RDT).

### Laboratory testing algorithm

Acute samples were tested using a commercial RDT for dengue NS1 antigen and IgM/IgG (Dengue Duo, Standard Diagnostics, Yongin-Si, Korea) on the first visit at the facility (day 1). All acute and convalescent samples were subsequently tested in the KEMRI laboratory using commercial dengue IgM/IgG ELISA kits (SD Dengue IgM/IgG Capture ELISA, Standard Diagnostics, Yongin-Si, Korea). The results from IgM/IgG ELISA and RDT were used to select those samples that would be further tested. Those samples that met the following criteria underwent molecular analysis with RT-PCR (Alm et al., 2015): (i) NS1- and/or IgM-positive on the rapid test in the acute phase; and/or (ii) seroconverted between acute and convalescent phases on IgM and IgG capture ELISA. RT-PCR was also performed on a limited number of randomly selected samples that were seropositive at both acute and convalescent time points by IgM/IgG capture ELISA, as well as those seronegative by RDT and IgM/IgG ELISA at all time points. In addition, all convalescent samples were tested using commercial chikungunya IgM ELISA kit (SD Chikungunya IgM ELISA, Standard Diagnostics, Yongin-Si, Korea). The detailed laboratory testing procedure has been previously described (Lim et al., 2018).

Laboratory confirmation for dengue infection was performed according to WHO diagnostic criteria (World Health Organization, 2012). Sero-conversion of anti-dengue IgM and IgG between the acute and convalescent phases and/or virus detection (RT-PCR) in the acute serum specimen was considered to be confirmed dengue. A positive IgM serology in single serum and/or positive on NS1 or IgM of RDT in single acute serum were criteria for probable dengue infection (World Health Organization, 2012). Confirmed and probable dengue infections were grouped to be dengue-positive in this analysis. Samples with negative results on RT-PCR and seronegative results on paired IgM/IgG ELISA results were classified as non-dengue. A positive IgG serology in a single serum, with negative results from all other tests, was classified as non-dengue.

### Statistical analysis

A descriptive summary of characteristics is presented for dengue-positive versus non-dengue cases. Body temperature ≥38.0 °C, the 75th percentile of the body temperature measured at the time of enrollment, was used to create a dichotomous variable (i.e., <38.0 °C vs. ≥ 38.0 °C). Clinical diagnosis at admission, prior to lab-confirmation, was categorized as suspected dengue, undifferentiated fever, and non-dengue. Yellow fever vaccination history was dichotomized between those who reported having been vaccinated versus those who did not remember or reported no vaccination.

In April 2017, we observed a steep rise in the dengue caseload in the study facilities. Mombasa County health officials issued a public health alert over a dengue outbreak, and it was declared an outbreak in May (Impouma, 2017, Githeko, 2017, Sanga, 2017, Onsarigo, 2017). The last two months of surveillance were grouped as an outbreak period and the previous months as non-outbreak. Categorical comparisons were made across dengue status using χ^2^ or Fisher’s exact tests. Continuous variables were compared using Student’s *t*-test or ANOVA. All analyses were performed using SAS version 9.4 (SAS Institute, Cary, North Carolina).

### Ethical considerations

The study protocol obtained ethical approval from the Institutional Review Boards (IRBs) of the International Vaccine Institute and the London School of Hygiene and Tropical Medicine, the ethical review committee of CPGH, and the KEMRI Scientific and Ethical Review Unit.

## Results

### General characteristics of subjects

Of 513 enrolled individuals, 31 had incomplete visit 1 (acute) lab data (i.e., RDT results available but no sample for ELISA or PCR, Figure 2). These patients were similar to those in the analysis sample in terms of age, gender, days into illness at the time of enrollment, and whether or not they were kept under observation.

The analysis sample includes 482 patients. Close to 80% of the dengue-positive patients were between 15 and 34 years (Table 1).

**Table 1:** Demographic and clinical characteristics between dengue-positive and non-dengue cases among febrile enrollees of the health facility-based fever surveillance in Mombasa, Kenya in 2016-2017.

Characteristics	Dengue-positive (n = 295) No. (%)*	Non-dengue (n = 187) No. (%)*	Total (n = 482) No. (%)*	p-value
Place of enrollment				0.645
CPGH	139 (47.12)	94 (50.27)	233 (48.34)	
Tudor	123 (41.69)	70 (37.43)	193 (40.04)	
Ganjoni	33 (11.19)	23 (12.30)	56 (11.62)	
Mean age (SD)	23.35 (9.23)	23.14 (13.46)	23.27 (11.05)	0.839
Age group (years)				**<.001**
1−4	8 (2.71)	31 (16.58)	39 (8.09)	
5−9	10 (3.39)	6 (3.21)	16 (3.32)	
10−14	13 (4.41)	6 (3.21)	19 (3.94)	
15−19	45 (15.25)	21 (11.23)	66 (13.69)	
20−24	124 (42.03)	39 (20.86)	163 (33.82)	
25−34	61 (20.68)	44 (23.53)	105 (21.78)	
35−44	24 (8.14)	28 (14.97)	52 (10.79)	
45−55	10 (3.39)	12 (6.42)	22 (4.56)	
Female	117 (39.66)	90 (48.13)	207 (42.95)	0.067
Required observation/Outpatients	2 (0.68)/293 (99.32)	0/187 (100.0)	2 (0.41)/480 (99.59)	0.259
Fever duration prior to visit (mean days, SD)	2.96 (1.92)	2.84 (1.79)	2.91 (1.87)	0.513
Fever duration, entire illness (mean days, SD)**	6.88 (3.75)	4.91 (2.76)	6.17 (3.55)	**<.001**
Mean temperature at presentation (SD)	37.85 (0.66)	37.71 (0.73)	37.80 (0.69)	**0.024**
Temperature at presentation				**0.014**
Below 38.0°c	179 (60.68)	134 (71.66)	313 (64.94)	
≥38.0°c	116 (39.32)	53 (28.34)	169 (35.06)	
Prev. dengue infection	3 (1.02)	3 (1.60)	6 (1.24)	0.323
YF vaccination	146 (49.49)	77 (41.18)	223 (46.27)	0.074
Clinical diagnosis				
Suspected dengue	186 (63.05)	18 (9.63)	204 (42.32)	**<.001**
Undifferentiated fever	76 (25.76)	121 (64.71)	197 (40.87)	
Non-dengue	33 (11.19)	48 (25.67)	81 (16.80)	
URI	18 (54.55)	27 (56.25)	45 (55.56)	
Malaria	1 (3.03)	3 (6.25)	4 (4.94)	
UTI	2 (6.06)	2 (4.17)	4 (4.94)	
Others	12 (36.36)	16 (33.33)	28 (34.57)	
Signs and symptoms (presence)				
Rash	34 (11.53)	10 (5.35)	44 (9.13)	**0.022**
Fatigue/weakness	269 (91.19)	156 (83.42)	425 (88.17)	**0.010**
Headache	282 (95.59)	155 (82.89)	437 (90.66)	**<.001**
Retro-orbital pain	166 (56.27)	69 (36.90)	235 (48.76)	**<.001**
Neck pain	90 (30.51)	43 (22.99)	133 (27.59)	0.072
Ear pain	23 (7.80)	10 (5.35)	33 (6.85)	0.300
Nasal congestion	15 (5.08)	26 (13.90)	41 (8.51)	**0.001**
Rhinorrhea	27 (9.15)	37 (19.79)	64 (13.28)	**0.001**
Sore throat	17 (5.76)	22 (11.76)	39 (8.09)	**0.019**
Cough	46 (15.59)	48 (25.67)	94 (19.50)	**0.007**
Sputum production	9 (3.05)	15 (8.02)	24 (4.98)	**0.015**
Nausea & vomiting	151 (51.19)	75 (40.11)	226 (46.89)	**0.018**
Diarrhea	31 (10.51)	25 (13.37)	56 (11.62)	0.340
Constipation	13 (4.41)	9 (4.81)	22 (4.56)	0.835
Abdominal pain	101 (34.24)	55 (29.41)	156 (32.37)	0.270
Nose bleeding	8 (2.71)	0	8 (1.66)	**0.026**
Gum bleeding	10 (3.39)	0	10 (2.07)	**0.008**
Flushed face	6 (2.03)	5 (2.67)	11 (2.28)	0.647
Loss of appetite	195 (66.10)	93 (49.73)	288 (59.75)	**<.001**
Myalgia	221 (74.92)	114 (60.96)	335 (69.50)	**0.001**
Arthralgia	222 (75.25)	104 (55.61)	326 (67.63)	**<.001**

* unless noted otherwise.

** only among those that reported the end of fever illness (n = 309; 199 dengue and 110 non-dengue patients).

### Laboratory-based confirmation of dengue cases

The breakdown by 3-level dengue status (dengue-confirmed, probable, and non-dengue) is presented in the supplementary table (table S1). Table 1 describes the demographic and clinical characteristics between non-dengue and dengue-positive cases with dengue-confirmed and probable combined (i.e., the 2-level status). Of 482 patients in the analysis sample, 46% (n = 223) had confirmed dengue infections based on paired ELISA and/or PCR. There were 15% (n = 72) classified as dengue-probable, based on RDT and/or ELISA seropositivity, and 39% (n = 187) as non-dengue cases (Figure 2). Of these dengue-positive cases, 69% (205/295) were based on PCR confirmation (Figure 2). Also, 28% (48 of 173 paired samples tested) and 24% (40 of 167 paired samples tested) were lab-confirmed with dengue infection by seroconversion between acute and convalescent samples using IgM and IgG capture ELISA. There were 32 patients confirmed by both PCR and ELISA (either IgM or IgG seroconversion) and 18 patients by seroconversion on ELISA alone.

Of the 482 RDT results, 39% (n = 189) were positive for NS1 and/or IgM. In terms of clinical diagnosis, 63% (186/295) of dengue-positive patients had clinically suspected dengue prior to lab-confirmation.

We retrospectively performed chikungunya IgM ELISA tests on all the convalescent samples; none were found to be chikungunya positive.

### Dengue serotypes during the outbreak and non-outbreak periods

**Figure 3:**
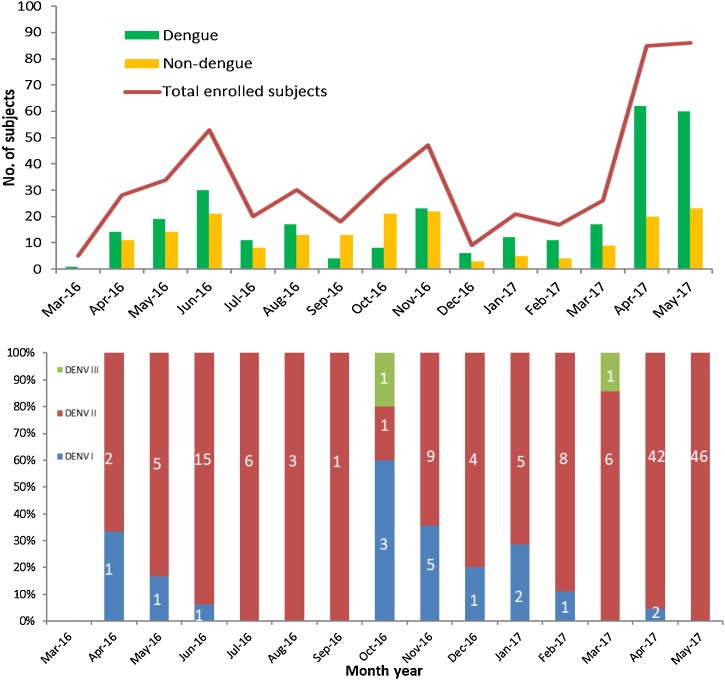
Monthly distribution of the enrolled febrile patients and patients by dengue status as well as serotype distribution. Notes: The figure has two parts. Panel A: monthly distribution of dengue-positive and non-dengue cases among the enrolled patients; Panel B: % of serotype composition (numbers shown in the bars) by month.

There were peaks of dengue incidence in April-June 2016 and April-May 2017 (Figure 3), coinciding with the “long rains” season. Of 295 dengue cases in the analysis sample of 482 patients, 173 were identified before (173/317), and 122 during the outbreak (122/165). DENV-2 was the predominant serotype before and during the outbreak, with DENV-1 remaining at lower levels throughout the study period (Figure 3).

### Clinical characteristics of dengue cases

Only two of the 482 patients required observation, both for less than three days, and both were dengue-positive. Of the 482 patients, the average time between fever onset and presentation was 3.0 days, with no evidence that this differed between dengue-positive and non-dengue cases. However, the average entire duration of fever illness was significantly longer for dengue cases than non-dengue cases — 6.9 versus 4.9 days — among 309 patients with data on the endpoint of their fever illness duration.

Of the non-dengue patients, 64.7 and 25.7% were clinically diagnosed with undifferentiated fever and non-dengue, respectively. Regarding symptoms, rash, fatigue, headache, retro-orbital pain, nausea/vomiting, nose bleeding, gum bleeding, loss of appetite, myalgia, and arthralgia were found more commonly among dengue-positive cases, compared to non-dengue.

## Discussion

Overall, evidence about dengue in Africa is limited (Amarasinghe et al., 2011) and is mostly from outbreak investigations and retrospective testing of existing serum banks from other studies (Ellis et al., 2015, Obonyo et al., 2018), although, among African countries, Kenya has relatively more evidence (Ellis et al., 2015, Obonyo et al., 2018, Konongoi et al., 2016, Masika et al., 2020, Inziani et al., 2020, Ochieng et al., 2015). Our data showed that dengue infection is an important cause of non-malarial febrile illness in patients seeking care at public health facilities and adds to the available literature on the endemicity of dengue in Mombasa.

### General and clinical characteristics of dengue cases

Our study’s key finding was that a substantial number of dengue-positive cases was identified in Mombasa, during an outbreak in Apr/May 2017, as well as the notable baseline caseload even in the non-outbreak period. Of 482 non-malarial febrile patients, 61.2% were identified as dengue-positive. Of those patients enrolled before the outbreak, approximately half (54.6%) were dengue-positive compared to more than two-thirds during the outbreak (73.9%). Shown in Figure 3 as a steep slant in caseload, our study was only able to capture the start of the outbreak in April and May; an alert over the outbreak was first reported by media in early May (Impouma, 2017, Sanga, 2017, Onsarigo, 2017). Officially reported to WHO, although in May and June 2017, the estimate was similar to our study; more than half of the individuals included (540/945) were lab-confirmed with dengue during this outbreak in Mombasa (World Health Organization, 2017).

Among comparable previous studies, few reported levels of dengue as high as this study (Obonyo et al., 2018). In particular, a hospital-based surveillance study conducted in Mombasa in 2013 found that, among 267 cases with suspected dengue, 58% were lab-confirmed with a current infection (Ellis et al., 2015). In our data, of 204 dengue-suspected patients, 156 (76%) were dengue-confirmed by either PCR and/or ELISA (suppl. Table 1). There may be differences in study settings, but these still indicate similarly high proportions of dengue cases.

On the other hand, most other published findings differ from ours. One study reported 15% of 500 febrile patients identified in CPGH from January 2014 to March 2015 to be DENV-confirmed, based on in-house indirect ELISA and Focus Reduction Neutralization tests (FRNT), even without screening out malaria RDT-positive cases (Munyuga et al., 2016). A study conducted in Kilifi, on the coast 70 km north of Mombasa, reported that 10% of febrile adults who had neither acute HIV infection nor malaria (by RDT) were PCR-confirmed with dengue in 2014–2015 (Ngoi et al., 2016). Among the corresponding subgroup in our study, 173 (50.4%) dengue cases were confirmed by either PCR and/or ELISA among 343 febrile patients between 18 and 35 years old. Even if we consider the differences in the study setting and methods, the estimate of the proportion with dengue was higher in our data.

During the study period, there was a programmatic challenge to patient recruitment due to a strike among medical officers for 71 days between September and November of 2016. Health facilities remained operational with other staff, but the official absence of medical officers at facilities could have influenced health-seeking behavior, and our surveillance might otherwise have enrolled a larger volume of febrile patients. If so, our denominator of non-malarial fever cases could have been bigger, and the proportion of dengue before the outbreak could have been lower.

Chikungunya virus was suspected to be one of the possible co-circulating pathogens in the area (Heath et al., 2020). To seek co-infections with dengue, we retrospectively performed chikungunya IgM ELISA tests on all the convalescent samples; none was found positive. Furthermore, our study did not enroll malaria RDT-positive patients in the screening process. This may be a limitation to the study, as some of them could have had co-infection with dengue. The review by Stoler et al. also highlighted the overdiagnosis of malaria among febrile episodes and supported that more attention should be given to dengue in West Africa, based on the available literature (Stoler et al., 2014). However, a study of the 2011 dengue outbreak in Mandera town, Kenya, reported four out of 30 lab-confirmed dengue cases to have malaria co-infection (Obonyo et al., 2018). In 2014–2015, a study tested sera from 385 febrile children in four study sites in Kenya using microscopy and real-time molecular assays for DENVs, chikungunya virus, malaria, and *Leptospira* (Waggoner et al., 2017). While 15 patients had coinfections- with *P. falciparum* and CHIKV, none had co-infection with malaria and dengue (Waggoner et al., 2017). Also, Amoako et al. reported one case of malaria and dengue co-infection out of 166 children with AFI in Ghana, where malaria is the predominant disease (Amoako et al., 2018). Overall, based on the available literature, such concurrent infection is reported to be uncommon (Wiwanitkit, 2011, Epelboin et al., 2012).

In terms of symptoms, dengue cases were associated with rash, fatigue, headache, retro-orbital pain, nausea/vomiting, nose bleeding, gum bleeding, loss of appetite, myalgia, and arthralgia, compared to non-dengue. These were also reported as being positively associated with dengue in other studies, including a surveillance study of the 2011 dengue outbreak in Mandera, Kenya (Obonyo et al., 2018, Guo et al., 2017, Humayoun et al., 2010, Hotchandani, 2014, Medina et al., 2011, Low et al., 2006, Ahmed et al., 2008).

### Dengue serotypes during outbreak and non-outbreak periods

In our study covering 2016–2017, DENV-2 was the predominant serotype both before and during the outbreak (Figure 3). A study of febrile patients in CPGH from January 2014 to March 2015 also reported DENV-2 as the predominant serotype, followed by DENV-3 and DENV-1 (Munyuga et al., 2016), as did a study of the outbreaks in 2013–2014 and 2017 based on sequencing results (Langat et al., 2020). Although not observed in this study, outbreaks may coincide with a shift in dengue serotypes (Bennett et al., 2010, Gubler, 1998, Saha et al., 2016), and earlier reports, covering the times prior to DENV-2 in circulation, support DENV-1 to be the prevalent serotype in Kenya. In Mombasa, between 2011 and 2014, including the 2013 outbreak, the most frequent serotype was DENV-1, followed by DENV-3 (Konongoi et al., 2016, Koech, 2015). DENV-1 was the prevalent serotype in circulation in March and April 2016 in a cohort of children in Western Kenya, in Kisumu and Chulaimbo (Vu et al., 2017). DENV-2 may have partially replaced DENV-1 prior to our study. However, it is difficult to determine whether there were virological differences between outbreak and non-outbreak periods without detailed information on virus strain.

### Mild case profile

Most of our dengue-positive cases were mild. Only two cases required observation for two days (discharged the day after admission), and both were dengue-positive. Both were clinically diagnosed with dengue, not DHF, and no complications were recorded. Our study did not collect data on other indicators of dengue severity. No patient reported hemorrhagic signs, with few patients reporting warning signs of severe dengue.

Although disease severity may be associated with secondary dengue infection, due to small numbers, this study could not draw meaningful conclusions in clinical differences between primary and secondary dengue cases. Among 163 cases with both IgM and IgG ELISA results available on paired sera, ten cases, all outpatients with ages ranging from 14 to 42 years, were likely secondary infections based on IgM seroconversion from acute to convalescent samples with IgG seropositivity (Cordeiro et al., 2009).

Clinical responses depend on several factors (Halstead, 2019), including age and exposure to a heterotypic virus. It is commonly reported for dengue patients to have outcomes with warning signs or severe dengue, and often they are associated with young age (<10 years) (Burattini et al., 2016, Halstead, 2006) or among the elderly population with underlying diseases (Huy et al., 2019) in other known dengue-endemic regions. However, there are limited data from Kenya and Africa on how dengue affects different age groups.

Among those attending the study facilities in this study, dengue cases were concentrated between 15–34 years of age, and this reflects the enrollment rate also being concentrated in the same age groups and the age structure of the population. About 45% of Mombasa residents are aged between 10–29 years, 31% over 30 years, and about 24% under ten years (ICT Authority, 2009). Nonetheless, the observed high proportion of dengue-positive cases in teenagers and young adults was higher on average than in the 2011 outbreak in Mandera, in which 30% of dengue cases were under ten years old with another 20% between 10–19 years (Obonyo et al., 2018). This was also consistent with data from Sudan, in which 73% of clinically diagnosed DHF cases in 2005 were between 5–15 years of age (Malik et al., 2011). However, given the youth of Mombasa’s age structure, our data, based on enrollment skewed to a younger population, are not sufficient to determine dengue epidemiology patterns with respect to the age, generalizable to the entire country.

The mildness of dengue disease may be due to protective genetic variants (Jaenisch et al., 2014). There is evidence from Cuba that the expression of specific genes is associated with severity (Sierra et al., 2017). Although our study did not seek to record race or ethnicity, 95% of Kenyans self-identify with an ethnic group associated with the Nilo-Saharan (Nilotic), Cushitic or Bantu language families (Kenya national bureau of statistics, 2013, Campbell and Tishkoff, 2008). Bantu ethnic groups are likely to be genetically closer to the West African ancestors of most Afro-Cubans (Sierra et al., 2017). Since there is a considerable genetic variation between Africans (Yu et al., 2002), either the same or other genes may be responsible for the lower risk of dengue in black Africans observed in Tanzania (Boillat-Blanco et al., 2018). In any case, such genetic factors, associated with race, may help explain our findings of dengue as a mild disease in native residents of Mombasa.

While there are data supporting a reduced risk of severe dengue in Africa (Jaenisch et al., 2014, Boillat-Blanco et al., 2018), there have been reports of severe dengue cases. During a dengue epidemic in the urban parts of Senegal in 2009, 196 of 696 serum samples were dengue-confirmed; there were 31 hospitalizations, five DHF, and one fatal case of DSS (Faye et al., 2014). In Sudan, one paper reported 81 IgM-based dengue-confirmed patients in 2010, classifying 58% of them as DHF and 11% as DSS using the WHO criteria (Abdallah et al., 2012). Another paper based on the 2005 DHF outbreak in Sudan reported 312 cases hospitalized with clinically diagnosed DHF with 11.9% DSS and a 3.8% mortality rate among patients, with most between 5–15 years of age (Malik et al., 2011). Nonetheless, this is largely understudied in Africa, with the currently available data focused on either Senegal or Sudan.

### Study limitations and strengths

Dengue transmission can vary substantially over time and space. In endemic areas, dengue epidemics occur at between three and 5-year intervals (Bennett et al., 2010). Hence, the generalizability of our study is limited by its duration of 15 months and geographical restriction to one area of the Mvita sub-county. Furthermore, one source of bias could be due to the study design, where cases were enrolled only at our study facilities, and we missed those community residents seeking care from healthcare providers other than the study facilities, including private clinics. This may further restrict the generalizability of the findings.

Nonetheless, our study held several strengths. By implementing the surveillance at three different KEPH levels of public health facilities, we were able to capture patients seeking care at various levels of healthcare service. Unlike previous reports in Kenya focusing mostly on outbreaks, this study captured the time before the outbreak as well as the first two months of the outbreak, with a large sample size and high dengue caseload, enabling an exploration of the differences between dengue and non-dengue cases (Figure 3) (Impouma, 2017, Sanga, 2017, Onsarigo, 2017).

## Conclusion

Our data provide evidence for a high level of transmission of dengue in Mombasa and demonstrate the magnitude of the dengue caseload during the 2017 outbreak and during the non-outbreak period. Almost all of our dengue cases were mild. Nonetheless, given the repeated outbreaks and endemicity of DENV transmission in Kenya, there should be improved case detection, clinical diagnosis in the clinical setting, and strengthened monitoring of dengue outbreaks. Furthermore, more data are needed to document clinical and epidemiologic patterns of dengue in Africa, which may differ from those in Southeast Asia and the Americas.

## Contributor’s statement page

All persons designated as authors have participated sufficiently in this work to take public responsibility for appropriate portions of the content. All the authors contributed in some or all areas of acquisition of funding, conception of the study, collection of data, analysis, and interpretation of data, drafting the article, article revision, scientific support, and final approval of the version to be published. The authors meet the criteria for authorship and qualify for authorship of this manuscript.

## Declaration of interest

I certify that the authors do not have any relevant financial relationships or potential conflicts of interest to disclose regarding the material discussed in this manuscript.

## Financial disclosure

This study was supported by funding from the 10.13039/100000865Bill and Melinda Gates Foundation (grant #: OPP 1053432) and the governments of Sweden, India, and the Republic of Korea. TE and NA were supported by award MR/R010161/1, which is jointly funded by the UK Medical Research Council (MRC) and the UK Department for International Development (DFID) under the MRC/DFID Concordat agreement and is also part of the EDCTP2 programme supported by the European Union.

The funders had no role in study design, data collection, analysis, decision to publish, or manuscript preparation.
