# Supplementary material for: Clinical and epidemiologic characteristics associated with dengue fever in Mombasa, Kenya
Source: Int J Infect Dis. 2020 Nov;100:207–15. doi: 10.1016/j.ijid.2020.08.074 (PMC7670221; doi:10.1016/j.ijid.2020.08.074)
Supplement: Supplementary file 1 [file mmc1.docx]

Table S1. Demographic and clinical characteristics by dengue confirmation status among febrile enrollees of the health facility-based fever surveillance in Mombasa, Kenya in 2016-2017

| Characteristics | Dengue-confirmed  (n=223) | Dengue-probable (n=72) | Non-dengue  (n=187) | Total  (n=482) | p-value |
| --- | --- | --- | --- | --- | --- |
| Place of enrollment |  |  |  |  | 0.215 |
| CPGH | 97 (43.50) | 42 (58.33) | 94 (50.27) | 233 (48.34) |  |
| Tudor | 100 (44.84) | 23 (31.94) | 70 (37.43) | 193 (40.04) |  |
| Ganjoni | 26 (11.66) | 7 (9.72) | 23 (12.30) | 56 (11.62) |  |
| Mean age (SD) | 23.04 (8.28) | 24.29 (11.71) | 23.14 (13.46) | 23.27 (11.05) | 0.694 |
| Age group (years) |  |  |  |  | **<.001** |
| 1-4 | 4 (1.79) | 4 (5.56) | 31 (16.58) | 39 (8.09) |  |
| 5-9 | 6 (2.69) | 4 (5.56) | 6 (3.21) | 16 (3.32) |  |
| 10-14 | 11 (4.93) | 2 (2.78) | 6 (3.21) | 19 (3.94) |  |
| 15-19 | 38 (17.04) | 7 (9.72) | 21 (11.23) | 66 (13.69) |  |
| 20-24 | 95 (42.60) | 29 (40.28) | 39 (20.86) | 163 (33.82) |  |
| 25-34 | 48 (21.52) | 13 (18.06) | 44 (23.53) | 105 (21.78) |  |
| 35-44 | 17 (7.62) | 7 (9.72) | 28 (14.97) | 52 (10.79) |  |
| 45-55 | 4 (1.79) | 6 (8.33) | 12 (6.42) | 22 (4.56) |  |
| Female | 87 (39.01) | 30 (41.67) | 90 (48.13) | 207 (42.95) | 0.173 |
| IPD/OPD | 1 (0.45)/222 (99.55) | 1 (1.39)/71 (98.61) | 0/187 (100.0) | 2 (0.41)/480 (99.59) | 0.296 |
| Fever duration prior to visit (Mean days, SD) | 2.96 (1.86) | 2.97 (2.12) | 2.84 (1.79) | 2.91 (1.87) | 0.806 |
| Fever duration, entire illness (Mean days, SD)* | 7.04 (3.78) | 6.28 (3.61) | 4.91 (2.76) | 6.17 (3.55) | **<.001** |
| Mean temperature at presentation (SD) | 37.87 (0.67) | 37.81 (0.63) | 37.71 (0.73) | 37.80 (0.69) | 0.064 |
| Temperature at presentation |  |  |  |  | **0.031** |
| Below 38.0°c | 132 (59.19) | 47 (65.28) | 134 (71.66) | 313 (64.94) |  |
| ≥ 38.0°c | 91 (40.81) | 25 (34.72) | 53 (28.34) | 169 (35.06) |  |
| Prev. dengue infection | 2 (0.90) | 1 (1.39) | 3 (1.60) | 6 (1.24) | 0.426 |
| YF vaccination | 115 (51.57) | 31 (43.06) | 77 (41.18) | 223 (46.27) | 0.092 |
| Clinical diagnosis |  |  |  |  |  |
| Suspected dengue | 156 (69.96) | 30 (41.67) | 18 (9.63) | 204 (42.32) | **<.001** |
| Undifferentiated fever | 44 (19.73) | 32 (44.44) | 121 (64.71) | 197 (40.87) |  |
| Non-dengue | 23 (10.31) | 10 (13.89) | 48 (25.67) | 81 (16.80) |  |
| URI (% of non-dengue) | 13 (56.52) | 5 (50.00) | 27 (56.25) | 45 (55.56) |  |
| Malaria | 1 (4.35) | 0 | 3 (6.25) | 4 (4.94) |  |
| UTI | 2 (8.70) | 0 | 2 (4.17) | 4 (4.94) |  |
| Pneumonia | 0 | 0 | 3 (6.25) | 3 (3.70) |  |
| Diarrheal illness | 1 (4.35) | 0 | 1 (2.08) | 2 (2.47) |  |
| Others | 6 (26.09) | 5 (50.00) | 12 (25.00) | 23 (28.40) |  |
| Signs and symptoms (presence) |  |  |  |  |  |
| Rash | 27 (12.11) | 7 (9.72) | 10 (5.35) | 44 (9.13) | 0.060 |
| Fatigue/weakness | 205 (91.93) | 64 (88.89) | 156 (83.42) | 425 (88.17) | **0.029** |
| Headache | 215 (96.41) | 67 (93.06) | 155 (82.89) | 437 (90.66) | **<.001** |
| Retro-orbital pain | 133 (59.64) | 33 (45.83) | 69 (36.90) | 235 (48.76) | **<.001** |
| Neck pain | 70 (31.39) | 20 (27.78) | 43 (22.99) | 133 (27.59) | 0.166 |
| Ear pain | 19 (8.52) | 4 (5.56) | 10 (5.35) | 33 (6.85) | 0.401 |
| Breathing difficulty | 1 (0.45) | 0 | 5 (2.67) | 6 (1.24) | 0.131 |
| Nasal congestion | 10 (4.48) | 5 (6.94) | 26 (13.90) | 41 (8.51) | **0.003** |
| Rhinorrhea | 18 (8.07) | 9 (12.50) | 37 (19.79) | 64 (13.28) | **0.002** |
| Sore Throat | 12 (5.38) | 5 (6.94) | 22 (11.76) | 39 (8.09) | 0.057 |
| Cough | 35 (15.70) | 11 (15.28) | 48 (25.67) | 94 (19.50) | **0.025** |
| Sputum production | 6 (2.69) | 3 (4.17) | 15 (8.02) | 24 (4.98) | **0.044** |
| Nausea & vomiting | 120 (53.81) | 31 (43.06) | 75 (40.11) | 226 (46.89) | **0.017** |
| Diarrhea | 25 (11.21) | 6 (8.33) | 25 (13.37) | 56 (11.62) | 0.509 |
| Constipation | 10 (4.48) | 3 (4.17) | 9 (4.81) | 22 (4.56) | 0.972 |
| Abdominal pain | 81 (36.32) | 20 (27.78) | 55 (29.41) | 156 (32.37) | 0.220 |
| Nose bleeding | 6 (2.69) | 2 (2.78) | 0 | 8 (1.66) | **0.041** |
| Gum bleeding | 9 (4.04) | 1 (1.39) | 0 | 10 (2.07) | **0.008** |
| Flushed face | 3 (1.35) | 3 (4.17) | 5 (2.67) | 11 (2.28) | 0.287 |
| Loss of appetite | 155 (69.51) | 40 (55.56) | 93 (49.73) | 288 (59.75) | **<.001** |
| Myalgia | 169 (75.78) | 52 (72.22) | 114 (60.96) | 335 (69.50) | **0.004** |
| Arthralgia | 171 (76.68) | 51 (70.83) | 104 (55.61) | 326 (67.63) | **<.001** |

*only among those that reported the end of fever illness (n=309; 156 dengue-confirmed, 43 dengue-probable, and 110 non-dengue cases)
